# All optical dual stage laser wakefield acceleration driven by two-color laser pulses

**DOI:** 10.1038/s41598-018-30095-4

**Published:** 2018-08-06

**Authors:** Vishwa Bandhu Pathak, Hyung Taek Kim, J. Vieira, L. O. Silva, Chang Hee Nam

**Affiliations:** 1 Center for Relativistic Laser Science, Institute for Basic Science (IBS), Gwangju, 61005 Korea; 2 Advanced Photonics Research Institute, Gwangju Institute of Science and Technology (GIST), Gwangju, 61005 Korea; 3 GoLP/Instituto de Plasmas e Fusão Nuclear, Instituto Superior Técnico, Universidade de Lisboa, Lisbon, Portugal; 4 Department of Physics and Photon Science, GIST, Gwangju, 61005 Korea

## Abstract

We propose an all-optical dual-stage laser wakefield acceleration (LWFA), staged with co-propagating two-color laser pulses in a plasma medium, to enhance the electron bunch energy. After the depletion of the leading fundamental laser pulse that initiates self-injection and sets up the first stage particle acceleration, the subsequent second-harmonic laser pulse takes over the acceleration process and accelerates the electron bunch in the second stage over a significantly longer distance than in the first stage. In this all optical dual-stage LWFA, the electrons can gain 3 times higher energy as compared to the energy gain from the single stage LWFA driven by a single-color laser pulse with equivalent energy. Our multi-dimensional particle-in-cell simulations demonstrate that a 10-GeV electron bunch with 20-pC charge can be obtained by the two-color dual-stage LWFA using total input laser power of 0.6 PW.

## Introduction

Laser-plasma electron acceleration^1–5^ is a promising approach to the next generation of electron accelerators because it can provide an accelerating field that can be more than three orders of magnitude larger than those of conventional devices. The progress of high power laser technologies^6,7^ and theoretical expectations^8,9^ have stimulated conceptual designs to realize an electron accelerator with energy over 100 GeV based on laser wakefield acceleration (LWFA)^10^. The radial ponderomotive force associated with a short and intense laser pulse can lead to the cavitation of all plasma electrons from the region where the laser pulse propagates, creating a spherical plasma wave (bubble or blowout^11,12^). The large accelerating field associated with the blowout can trap and accelerate plasma electrons (self-injection), or positrons if a Laguerre-Gaussian laser beam is used as a driver^13^.

In LWFA, the acceleration length is one of the important parameters determining the final electron energy. For a self-guided laser pulse^14^, the effective acceleration length is limited either by the laser etching (depletion) length [L_etch_ ≈ (ω_0_/ω_p_)^2^cτ_L_]^9^, or by the dephasing length [${{\rm{L}}}_{{\rm{d}}}=(4/3)({{\rm{\omega }}}_{0}^{2}/{{\rm{\omega }}}_{{\rm{p}}}^{2})\sqrt{{{\rm{a}}}_{0}}\,{\rm{c}}/{{\rm{\omega }}}_{{\rm{p}}}$]^9^, where a_0_ = eE_0_/(m_e_cω_0_) is the normalized vector potential of the laser, c is the speed of light in vacuum, and E_0_, *ω*_0_ and τ_L_ are the peak laser electric field, laser central frequency and laser pulse length respectively. In addition, $\,{\omega }_{p}=\sqrt{4\pi {n}_{0}{e}^{2}/m}$ is the plasma frequency with *n*_0_ being plasma density, and *m*, *e* electron rest mass and elementary charge, respectively. As it is clear from the expressions above, the acceleration length, and thus the energy gain, is higher at lower plasma densities because the ratio ω_0_/ω_p_ is higher. In contrast, particle trapping is easily met at higher plasma densities, because the required longitudinal electron momentum for self-injection also scales with ω_0_/ω_p_. Therefore, the electron injection and the acceleration length are the competing factors in the optimization of plasma density for LWFA in single stage.

In order to solve this problem, dual-stage LWFA^10,15–19^ has been proposed with a short, high density plasma in the first stage (injector stage) and a long, low density plasma in the second stage (accelerator stage). The dual-staged LWFA can be achieved either by a single laser pulse propagating through two plasma media^16,20–22^ or by two independent laser pulses focused separately to two plasma media^15,18,19^. All these methods for dual-staged LWFA require two plasma media with Luo *et al*.^19^ showing multi stage coupling of LWFA in curved plasma channel from 2D PIC simulations using OSIRIS^23,24^.

Manipulating the laser frequency to realize a multi-stage LWFA is considerably more challenging. There are growing interests on all-optical control of LWFA, by manipulating optical properties of driving laser pulse^25–27^ or by composing multiple laser pulses^28–31^. While the frequency chirp approach can be employed to control injection and acceleration^27^, it is severely limited by the maximum chirp that may be introduced in a single laser pulse driver. Here we explore an innovative solution to this limitation by proposing to use a two-color laser pulse to create the injector and accelerator stage in a single homogeneous plasma column. In our proposed model, the leading pulse, at the fundamental frequency, acts as an injector and the trailing laser pulse, at the second harmonic, works as an accelerator or booster. We derived an analytical expression for the energy scaling of all-optical dual stage LWFA in the matched regime^9,12^. Using PIC simulations with OSIRIS^23,24^, we demonstrate the proof-of-principle of this concept to achieve quasi-mono-energetic multi-GeV electron bunch in 3D calculations and the generation of 10-GeV electron beam by two-color laser pulses having the input laser power of 0.6 PW in 2D calculations.

## Results

### Energy gain in all optical dual-stage LWFA

The two-color dual-stage LWFA can be understood as following: In the injector stage, the leading lower frequency laser pulse excites a plasma wave with a lower phase velocity, which triggers electron self-injection (injector stage). The second harmonic laser pulse, trailing just behind the leading pulse, is guided within the bubble. The second harmonic laser will drive wakefields once the leading pulse depletes. These wakefields move faster than those of the injector stage, being ideal to boost the energy of the self-injected electrons. This is the acceleration stage. The transition from the injection stage to the acceleration stage is regulated by the temporal delay between the two color laser pulses. We show that, by fine-tuning the time delay between the two-color laser pulses, quasi-mono-energetic features can be obtained in the accelerated electron beam.

To estimate the energy gain of the two-color dual-stage LWFA, let us consider a fundamental frequency laser pulse (FL) with normalized vector potential ${{\rm{a}}}_{0({{\rm{\omega }}}_{0})}$, frequency ω_0_, spot-size ${{\rm{W}}}_{0({{\rm{\omega }}}_{0})}$ and pulse duration ${{\rm{\tau }}}_{{\rm{L}}({{\rm{\omega }}}_{0})}$ is propagating in a homogeneous plasma with density n_0_. The FL is followed by a second harmonic laser pulse (SL) with time delay t_d_ with respect to FL, normalized vector potential ${{\rm{a}}}_{0(2{{\rm{\omega }}}_{0})}$, frequency 2ω_0_, spot-size ${{\rm{W}}}_{0(2{{\rm{\omega }}}_{0})}$ and pulse duration ${{\rm{\tau }}}_{{\rm{L}}(2{{\rm{\omega }}}_{0})}$. The length of the first stage of LWFA led by the FL is limited to ${{\rm{L}}}_{{\rm{s}}1}={c{\rm{\tau }}}_{{\rm{L}}({{\rm{\omega }}}_{0})}{{\rm{\omega }}}_{0}^{2}/{{\rm{\omega }}}_{{\rm{p}}}^{2}$ because of localized etching^32^, which is also close to the dephasing length in the matched regime^9^. Assuming that the trailing SL does not significantly influence the bubble evolution in the first stage, the energy gain in the first stage is given by ${{\rm{\Delta }}E}_{{\rm{s}}1}=(\frac{2}{3}){{\rm{mc}}}^{2}{{\rm{a}}}_{0({{\rm{\omega }}}_{0})}{{\rm{\omega }}}_{0}^{2}/{{\rm{\omega }}}_{{\rm{p}}}^{2}$. Once the leading laser pulse is absorbed, the SL takes over the wakefield excitation in the accelerator stage characterized by 4 times longer etching length as compared to the first stage. Since the acceleration length in the second stage is expected to be limited by the dephasing, the acceleration length of the second stage can be given as $\,{{\rm{L}}}_{{\rm{s}}2}=(8/3){{\rm{\omega }}}_{0}^{2}/{{\rm{\omega }}}_{{\rm{p}}}^{2}{\rm{R}}$, where ${\rm{R}}=2\sqrt{{{\rm{a}}}_{0(2{{\rm{\omega }}}_{0})}}$ ^9^ is the bubble radius. Hence, the energy gain in the second stage can be written as ${{\rm{\Delta }}E}_{{\rm{s}}2}=(8/3){\mathrm{mc}}^{2}{{\rm{a}}}_{0(2{{\rm{\omega }}}_{0})}{{\rm{\omega }}}_{0}^{2}/{{\rm{\omega }}}_{{\rm{p}}}^{2}$ and the total energy gain can be estimated as,

1 $${\rm{\Delta }}E=\frac{2}{3}{{\rm{mc}}}^{2}\frac{{{\rm{\omega }}}_{0}^{2}}{{{\rm{\omega }}}_{{\rm{p}}}^{2}}{{\rm{a}}}_{0({{\rm{\omega }}}_{0})}(1+4\frac{{{\rm{a}}}_{0(2{{\rm{\omega }}}_{0})}}{{{\rm{a}}}_{0({{\rm{\omega }}}_{0})}}).$$

For total input power ${{\rm{P}}}_{{\rm{in}}}={{\rm{P}}}_{{{\rm{\omega }}}_{0}}+{{\rm{P}}}_{2{{\rm{\omega }}}_{0}}$ with the FL power, ${{\rm{P}}}_{{{\rm{\omega }}}_{0}}$, the SL power, ${{\rm{P}}}_{2{{\rm{\omega }}}_{0}}$, and the second harmonic conversion efficiency ${\rm{\alpha }}={{\rm{P}}}_{2{{\rm{\omega }}}_{0}}/{{\rm{P}}}_{{\rm{in}}}$, we can re-write Eq. (1) as:

2 $${\rm{\Delta }}E[{\rm{GeV}}]=1.7\,{G}_{d}{(\frac{{{\rm{P}}}_{in}[{\rm{TW}}]}{100})}^{\frac{1}{3}}{(\frac{{10}^{18}}{{{\rm{n}}}_{0}[{{\rm{cm}}}^{-3}]})}^{\frac{2}{3}}{(\frac{0.8}{{{\rm{\lambda }}}_{0}[{\rm{\mu }}m]})}^{\frac{4}{3}},$$

where ${G}_{d}={(1-\alpha )}^{1/3}+{2}^{4/3}{\alpha }^{1/3}$ is the extra gain provided by the dual stage acceleration. For α = 0.05 to 0.9, G_d_ slowly varies from 2.0 to 3.0, providing a stable gain factor. For α = 0, *G*_*d*_ = 1 and the energy scales same as in Lu *et al*.^9^. However, for the same total laser input power our proposed scheme can significantly enhance the acceleration gain by a factor in the range between 2 to 3. As a result, the two-color laser pulses with P_in_ = 1 PW, nowadays available from a PW laser facility, can produce 10-GeV electron bunches for *α* = 0.3.

### Proof of principle simulation

In order to show the feasibility of the all-optical dual stage LWFA, a set of multi-dimensional PIC simulations are performed using OSIRIS^23,24^. We consider two-color laser pulses of input laser power 215 TW propagating in a 0.7-cm plasma with density 7.75 × 10^18^ cm^−3^. We initialize a simulation box, which moves with the speed of light c, has dimensions of 25 c/ω_p_ × 30 c/ω_p_ × 30 c/ω_p_ and are divided into 3000 × 280 × 280 cells, along z, x and y direction respectively, with 2 particles per cell. The FL with frequency ω_0_ = 15 ω_p_ is initialized with parameters: ${{\rm{a}}}_{0({{\rm{\omega }}}_{0})}=8$, ${{\rm{W}}}_{0({{\rm{\omega }}}_{0})}=6\,{\rm{c}}/{{\rm{\omega }}}_{{\rm{p}}}$ and ${{\rm{\tau }}}_{{\rm{L}}({{\rm{\omega }}}_{0})}=\frac{{\lambda }_{p}}{2}=3.14\,{{\rm{\omega }}}_{{\rm{p}}}^{-1}$, and the SL with frequency 2 ω_0_ and *α* = 0.3 is initialized in the simulation box at a distance 4.5c/ω_p_ away from the leading pulse with parameters ${{\rm{a}}}_{0(2{{\rm{\omega }}}_{0})}=4$, ${{\rm{W}}}_{0(2{{\rm{\omega }}}_{0})}=4\,{\rm{c}}/{{\rm{\omega }}}_{{\rm{p}}}$ and ${{\rm{\tau }}}_{{\rm{L}}(2{{\rm{\omega }}}_{0})}=\frac{{\lambda }_{p}}{2}$. The plasma is initialized just in front of the leading pulse, with an initial density ramp of size 100 c/ω_p_ and then a constant homogeneous plasma density.

**Figure 1:**
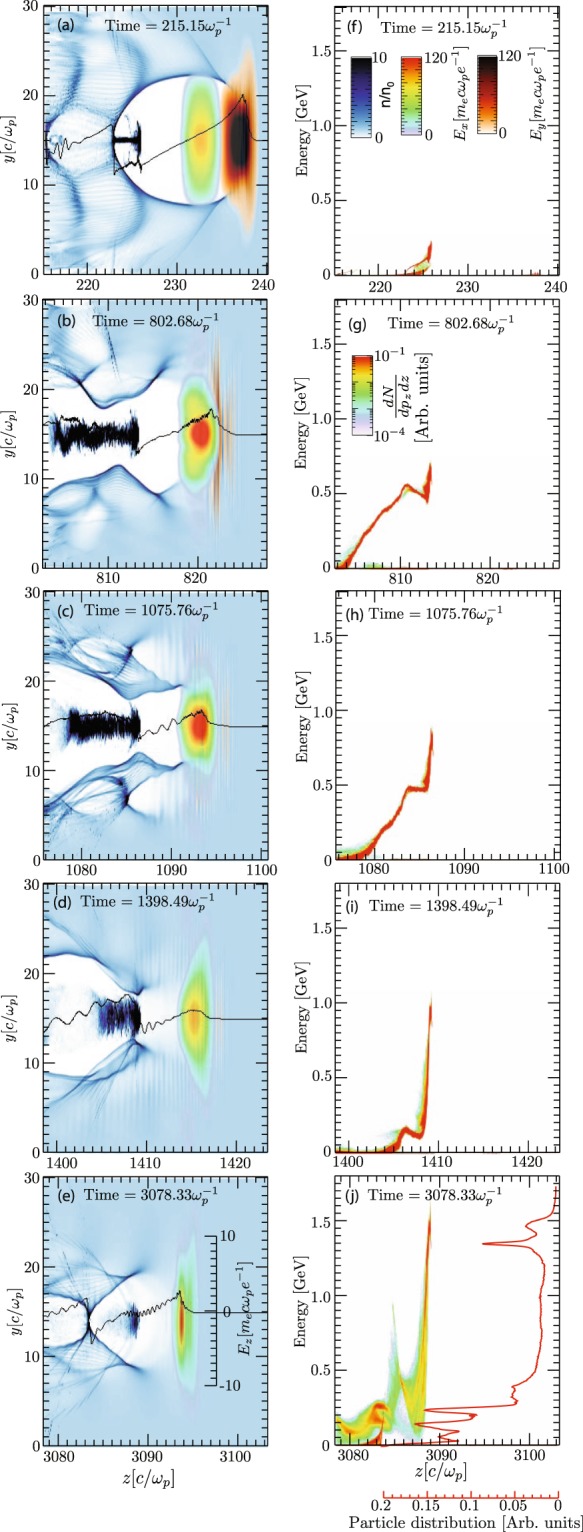
Proof-of-principle 3D PIC simulation. (**a**–**e**) Show the snapshots of laser fields and electron density distribution in the y-z plane at different times, and (**f**–**j**) show the energy-space distribution of accelerated electrons at respective times. The line in figures (**a**–**e**) is the longitudinal electric field (*E*_*z*_) on the laser axis. The red line in figure (**j**) represents energy spectrum of the electrons showing bunched electron beam around 1.4 GeV.

The 3D PIC simulations demonstrate that two-color laser pulses can realize the dual-stage acceleration. The results obtained from the 3D simulation is shown in Fig. 1, where panels (a)-(e) plot the laser fields for FL and SL, and the corresponding electron density distribution in the y-z simulation plane at various stages of the acceleration process, and panels (f)-(j) show the corresponding electron distribution in phase space. The panels (a) and (f) demonstrate the excitation of bubble, self-injection by FL, and the guiding of SL inside the bubble in the first stage. The panels (b) and (g) show the depletion of leading FL and the initial onset of the second stage by the SL. Panels (c) and (h) clearly show the coupling of the electron bunch, from the first stage to the second stage. An electron beam with 2nC charge in 500–600 MeV energy was injected into the second stage with 12mrad divergence and 16 mm mrad emittance, and further accelerated to 1.5 GeV with 0.3 nC charge above 1 GeV, as shown in panel (j). In electron energy spectrum we observe two quasi-mono-energetic peaks, one at 1.3 Gev with 2.4% full-width-half maximum energy spread, and another peak at 1.5 GeV with 6.7% energy spread. At the same time the beam divergence and emittance reduces to 4 mrad and 10 mm mrad respectively in the second stage. Thus, the proposed all optical staged LWFA has potential to increase the energy by a factor of 3, and reducing beam divergence and emittance, while the beam charge reduces. According to our analytical scaling [Eq. 2], the maximum energy gain for the total combined laser power of 215 TW, 800-nm fundamental wavelength and plasma density of 7.75 × 10^18^ cm^−3^, is 1.4 GeV that is very close to the final energy of 1.5 GeV obtained in the 3D PIC simulations, showing 3 times energy enhancement as compared to the energy obtained in the single stage LWFA. As clear from Fig. 1(e), the acceleration length in the second stage is limited by the dephasing of energetic electrons in the wake of the second stage and, as a result, quasi-mono-energetic electrons at 1.4 GeV are produced [Fig. 1: panel (j)].

In order to obtain the necessary condition for the coupling of electron beam to the second stage, the case of LWFA in the matched regime^9^ is considered with spherical and stationary nonlinear wakefields. The group velocity of the leading laser pulse is ${{\rm{v}}}_{{\rm{g}}1}\approx {\rm{c}}[1-(3/2)({{\rm{\omega }}}_{{\rm{p}}}^{2}/{{\rm{\omega }}}_{0}^{2})]$ because of localized etching^9,32^, and the following second harmonic laser pulse propagating within the first-stage bubble has group velocity v_g2_ ≈ c. As a result, by the end of the first stage the distance between the two pulses reduces to ${{\rm{L}}}_{{\rm{d}}}={{\rm{ct}}}_{{\rm{d}}}-(3/2){c{\rm{\tau }}}_{{\rm{L}}({{\rm{\omega }}}_{0})}$. During this time the leading part of the electron bunch, propagating with velocity c, also covers a distance ${{\rm{L}}}_{{\rm{b}}}=(\frac{3}{2}){c{\rm{\tau }}}_{{\rm{L}}({{\rm{\omega }}}_{0})}$ and, thus, the distance between the SL and the electron bunch stays constant in the first stage.

### Role of time delay on the acceleration

With these constraints, we can estimate the required time delay between FL and SL for the coupling. The lower limit of time delay is limited by two conditions; (i) for proper guiding SL must avoid the interaction with the remnant of the density pile-up created by the FL at its front; thus, ${{\rm{t}}}_{{\rm{d}}} > (\frac{3}{2}){{\rm{\tau }}}_{{\rm{L}}({{\rm{\omega }}}_{0})}$, and (ii) electrons accelerated in the first stage must reach the back of the bubble excited in the second stage, *i*.*e*. ${{\rm{L}}}_{{\rm{b}}}\ge 2({\rm{R}}({{\rm{\omega }}}_{0})-{\rm{R}}(2\,{{\rm{\omega }}}_{0}))-{{\rm{L}}}_{{\rm{d}}}$, which gives ${{\rm{t}}}_{{\rm{d}}}\ge 4(\sqrt{{{\rm{a}}}_{0({{\rm{\omega }}}_{0})}}-\sqrt{{{\rm{a}}}_{0(2{{\rm{\omega }}}_{0})}})/{{\rm{\omega }}}_{{\rm{p}}}$. The upper limit of time delay again can be obtained from two conditions: (i) at the time of injection in the first stage the electron should not interact with the tail of the SL to avoid degradation in the beam-emittance, *i*.*e*. ${{\rm{t}}}_{{\rm{d}}} < (4\sqrt{{{\rm{a}}}_{0({{\rm{\omega }}}_{0})}}-{{\rm{\tau }}}_{{\rm{L}}({{\rm{\omega }}}_{0})})/{{\rm{\omega }}}_{{\rm{p}}}$, and (ii) when coupled into the second stage the electrons should not already fall into the dephasing region of the second-stage-bubble, *i*.*e*. ${{\rm{t}}}_{{\rm{d}}} < 4(\sqrt{{{\rm{a}}}_{0({{\rm{\omega }}}_{0})}}-0.5\sqrt{{{\rm{a}}}_{0(2{{\rm{\omega }}}_{0})}})/{{\rm{\omega }}}_{{\rm{p}}}$. Thus, the suitable condition for choosing time delay for the coupling can be written as,

3 $$\begin{array}{c}{\rm{Min}}[4(\sqrt{{a}_{0({\omega }_{0})}}-0.5\sqrt{{a}_{0(2{\omega }_{0})}}),\,4\sqrt{{a}_{0({\omega }_{0})}}-{\tau }_{L(2{\omega }_{0})}]\\ \,\, > \,{\omega }_{p}{t}_{d} > \,{\rm{Max}}[1.5{\omega }_{p}{\tau }_{L({\omega }_{0})},4(\sqrt{{a}_{0({\omega }_{0})}}-\sqrt{{a}_{0(2{\omega }_{0})}})]\end{array}$$

Including the effect of time delay, the acceleration length of second stage modifies to ${{\rm{L}}}_{{\rm{s}}2}=(8/3)({{\rm{\omega }}}_{0}^{2}/{{\rm{\omega }}}_{{\rm{p}}}^{2})$ $(4\sqrt{{{\rm{a}}}_{0({{\rm{\omega }}}_{0})}}-2\sqrt{{{\rm{a}}}_{0(2{{\rm{\omega }}}_{0})}}-{{\rm{\omega }}}_{{\rm{p}}}{{\rm{t}}}_{{\rm{d}}})\,{\rm{c}}/{{\rm{\omega }}}_{{\rm{p}}}$, and thus the energy gain in the second stage can be given in details by ${{\rm{\Delta }}E}_{{\rm{s}}2}=(8/3){{\rm{mc}}}^{2}{{\rm{a}}}_{0(2{{\rm{\omega }}}_{0})}({{\rm{\omega }}}_{0}^{2}/{{\rm{\omega }}}_{{\rm{p}}}^{2})(2\sqrt{{{\rm{a}}}_{0({{\rm{\omega }}}_{0})}/{{\rm{a}}}_{0(2{{\rm{\omega }}}_{0})}}-{{\rm{\omega }}}_{{\rm{p}}}{{\rm{t}}}_{{\rm{d}}}/(2\sqrt{{{\rm{a}}}_{0(2{{\rm{\omega }}}_{0})}}))$.

**Figure 2:**
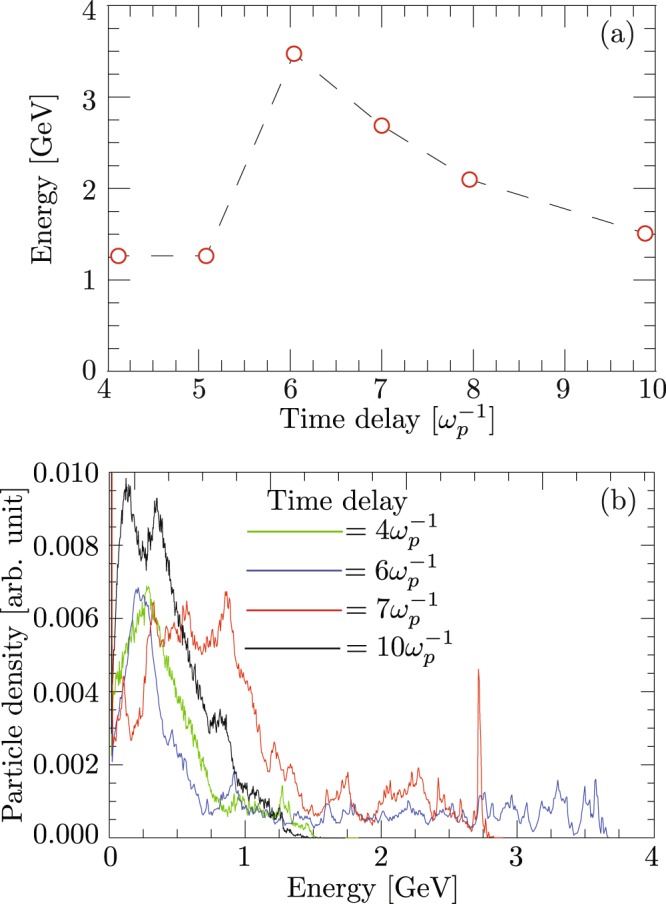
Effect of time-delay between the leading and trailing laser pulse on the electron energy. (**a**) Variation in the maximum energy with the time delay, and (**b**) spectrum of accelerated electrons by TFTP with time delay *ω*_*p*_*t*_*d*_ = 4 (green), 6 (blue), 7 (red), and 10 (black). The results are obtained with 2D PIC simulations.

To verify the role of time delay between the two pulses on the coupling of the electrons from the first stage into the second stage, we further perform a set of 2D simulations with two-color laser pulses of P_in_ = 210 TW and *α* = 0.33 propagating in a 1.5-cm plasma with density 4.35 × 10^18^ cm^−3^. The simulation box has dimensions of 25 c/ω_p_ × 40 c/ω_p_ and is divided into 4800 × 400 cells, along z and x direction respectively, with 9 particles per cell. We consider FL parameters as: ${{\rm{a}}}_{0({{\rm{\omega }}}_{0})}=5$, ω_0_ = 20 ω_p_, ${{\rm{W}}}_{0({{\rm{\omega }}}_{0})}=7\,{\rm{c}}/{{\rm{\omega }}}_{{\rm{p}}}$ and ${{\rm{\tau }}}_{{\rm{L}}({{\rm{\omega }}}_{0})}=\frac{{\lambda }_{p}}{2}$, and the SL parameters as: ${{\rm{a}}}_{0(2{{\rm{\omega }}}_{0})}=2.5$, ${{\rm{W}}}_{0(2{{\rm{\omega }}}_{0})}=5\,{\rm{c}}/{{\rm{\omega }}}_{{\rm{p}}}$ and ${{\rm{\tau }}}_{{\rm{L}}(2{{\rm{\omega }}}_{0})}=\frac{{\lambda }_{p}}{2}$. Though the laser parameters are not exactly in the matched regime^9^, the robustness of the proposed scheme can be tested. As shown in Fig. 2(a), the maximum energy can be optimized by manipulating the time delay between the two pulses. We observe that for *t*_*d*_ ≈ 1.8*τ*_*L*_ the peak energy of 3.6 GeV is obtained after the end of second stage. For smaller time delays our simulations showed no coupling of the accelerated electron from the first stage into the second stage. As we further increase the time delay between the two pulses the effective acceleration length and hence the energy of the trapped electrons in the second stage decreases due to dephasing, as evident from Fig. 2(a) for ${{\rm{t}}}_{{\rm{d}}} > \frac{6}{{{\rm{\omega }}}_{{\rm{p}}}}.$ We also perform a series of 2D PIC simulations to compare the dual stage LWFA driven by two single-frequency pulses (SFTP) with the dual stage driven by the two frequencies two pulse (TFTP). The details are provided in the supplementary material.

### 10 GeV electron beam driven by 0.6-PW laser

**Figure 3:**
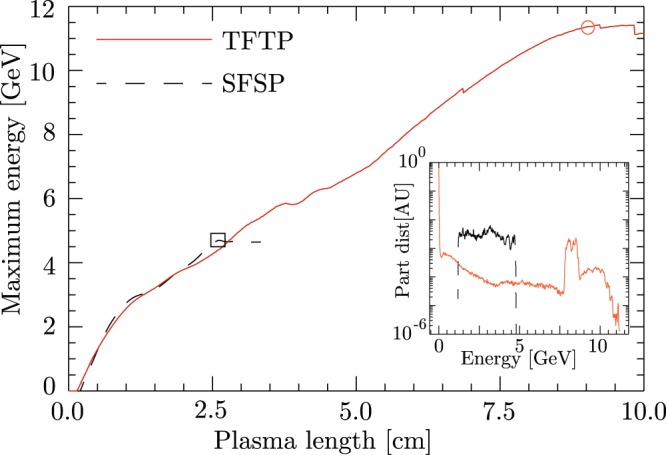
Time evolution of maximum energy of the accelerating electron bunch with 1PW laser. The solid line shows the energy gain from the TFTP, while the dashed line represents the energy gain from the single frequency single pulse (SFSP). The inset shows the electron spectra for the two cases at the plasma position of the maximum energy (⊙: *TETP*, ⊡: *SFSP*). The results are obtained with 2D PIC simulations.

As discussed before, one of the benefits of the two color dual stage LWFA is significant enhancement in the energy of an accelerated electron bunch, with Eq. (2) suggesting the realization of 10-GeV electron bunch. Keeping that in mind, we perform 2D PIC simulations using OSIRIS^23,24^, and the results are summarized in Fig. 3. We consider equally energetic FL and SL with total laser energy of 30 J and each with 50-fs pulse-length, which corresponds to P_in_ = 0.6 PW. The other FL and SL parameters are: ${{\rm{a}}}_{0({{\rm{\omega }}}_{0})}=5.38$, ω_0_ = 40 ω_p_, ${{\rm{W}}}_{0({{\rm{\omega }}}_{0})}=2\sqrt{{{\rm{a}}}_{0({{\rm{\omega }}}_{0})}}{\rm{c}}/{{\rm{\omega }}}_{{\rm{p}}}$, ${{\rm{\tau }}}_{{\rm{L}}({{\rm{\omega }}}_{0})}=\frac{{\lambda }_{p}}{2}$, ${{\rm{a}}}_{0(2{{\rm{\omega }}}_{0})}=3.4$, ${{\rm{W}}}_{0(2{{\rm{\omega }}}_{0})}=2\sqrt{{{\rm{a}}}_{0(2{{\rm{\omega }}}_{0})}}{\rm{c}}/{{\rm{\omega }}}_{{\rm{p}}}$, ${{\rm{\tau }}}_{{\rm{L}}(2{{\rm{\omega }}}_{0})}=\frac{{\lambda }_{p}}{2}$ and t_d_ = 4.5 /ω_p_. To ensure guiding of the SL for a long distance in the second stage, a parabolic plasma channel profile ${\rm{n}}={n}_{0}\,[1+(\frac{{{\rm{\Delta }}n}_{c}}{{{\rm{n}}}_{0}}){x}^{2}]$ is considered with ${\rm{\Delta }}{n}_{c}/{n}_{0}\,=(4/{{\rm{W}}}_{0(2{{\rm{\omega }}}_{0})}^{4}){(c/{{\rm{\omega }}}_{{\rm{p}}})}^{2}$ ^2,9^ and *n*_0_ = 1 × 10^18^ *cm*^−3^. The acceleration with a single laser pulse of energy 30 J and wavelength 0.8 μm is limited to 5 GeV (the dashed line in Fig. 3) after propagating 2.5-cm plasma due to dephasing. For the two-color dual-stage acceleration, the electron acceleration, after reaching 5 GeV in 2.5-cm plasma, is enhanced to 10 GeV with 20-pC charge in the second stage (the solid line in Fig. 3) within 8-cm plasma.

This is a significant improvement as compared to the single-stage LWFA. Lu *et al*.^9^ scaling in the matched regime indicates that to generate 10-GeV electron beam in a 10-cm plasma with density 6 × 10^17^ *cm*^−3^ a 10-PW laser pulse is required. On the contrary, in two-color dual-stage LWFA, if the guiding of the second harmonic laser pulse is insured, 10-GeV electron beam is obtained in the same plasma length with an order of magnitude lower laser power. On the other hand, to generate 10-GeV electron bunch in single stage LWFA at lower laser power of 1PW, the laser pulse needed to be externally guided through a 1-m long plasma channel with on-axis density of 2 × 10^17^ *cm*^−3^. Similarly, Vay *et al*.^33^ have also shown the possibility of obtaining 10-GeV electron bunch from a 40-J laser pulse, externally guided through a 1.5-m-long plasma channel with on-axis 1 × 10^17^ *cm*^−3^ plasma density. In comparison we have shown that two-color dual-stage LWFA can deliver same energies in one-order shorter plasma length. It is worth to mention the concerns on the transverse offset between the two pulse and plasma guiding structure. Such transverse offsets may lead to transverse oscillations of the laser in the plasma channel^34,35^, which can degrade the electron beam parameters, while it at the same can also lead to compact tunable polarized X-ray sources^35^.

## Conclusion

In conclusion, we have shown the feasibility of all optical two-color dual-stage LWFA with the help of multi-dimensional PIC simulations to obtain multi-GeV quasi-mono-energetic electron bunches. Two-color dual-stage LWFA yields higher acceleration energies, while keeping the quasi-mono-energetic property of the electron bunch, as compared to the same-energy single-pulse LWFA. We further provide estimates on the effect of the time delay between the two pulses on the acceleration process and show that, by fine-tuning the time delay and thus modifying the dephasing length in the second stage, the bunch energy and its quality can be optimized. We demonstrate that the two-color dual-stage LWFA can produce 10-GeV electron beam with an input power of 0.6 PW, which brings us closer to one of the milestones of LWFA.

## Electronic supplementary material

Supplementary information
