# Supplementary material for: All optical dual stage laser wakefield acceleration driven by two-color laser pulses
Source: Sci Rep. 2018 Aug 6;8:11772. doi: 10.1038/s41598-018-30095-4 (PMC6079035; doi:10.1038/s41598-018-30095-4)
Supplement: Supplementary file 1 — Supplementary information [file 41598_2018_30095_MOESM1_ESM.docx]

**Supplementary information for “All optical dual stage laser wakefield acceleration driven by two-color laser pulses”**

Vishwa Bandhu Pathak1, Hyung Taek Kim1,2, J. Vieira3, L. O. Silva3, Chang Hee Nam1,4

1 Center for Relativistic Laser Science, Institute for Basic Science (IBS), Gwangju 61005, Korea

2 Advanced Photonics Research Institute, Gwangju Institute of Science and Technology (GIST), Gwangju 61005, Korea

^3^GoLP/Instituto de Plasmas e Fusão Nuclear, Instituto Superior Técnico, Universidade de Lisboa, Lisbon, Portugal

and

4Department of Physics and Photon Science, GIST, Gwangju 61005, Korea

The single laser pulse of same energy evidently provides lower energy gain as compared to energy gain yield in the dual stage LWFA using FL and SL. The other critical question on the all-optical dual stage acceleration by two-color laser pulses can be what if both the lasers have same frequency, *i.e.* how dual stage LWFA driven by two single-frequency pulses (SFTP) compares with the dual stage driven by the two frequencies two pulse (TFTP). To answer this question, we performed multiple 2D PIC simulations by using various combinations of intensity ratios between the pulses in SFTP. In Fig. S1, we compare the time evolutions of maximum electron energy for cases : (i) TFTP with same parameters as in Fig. 2, (ii) SFTP with identical laser pulses ($a_{0(\omega_{0})}=4.34$, $W_{0(\omega_{0})}=7c/\omega_{p}$, $\tau_{L(\omega_{0})}=3\omega_{p}^{-1}$ and $\omega_{0}=20\omega_{p}$), (iii) SFTP with leading pulse $a_{0(\omega_{0})}=5.5$, trailing pulse $a_{0(\omega_{0})}=3.835$, and (iv) SFTP with leading pulse $a_{0(\omega_{0})}=6.0$, trailing pulse $a_{0(\omega_{0})}=1.85$. For the cases (iii) and (iv), rest of the laser parameters except intensities of the two pulses are same as in the case (ii). As shown in Fig. S1, TFTP has significant enhancement in the electron energy and compare to all the combinations of SFTPs. In the case (ii), electrons accelerated in the first stage already find itself in the dephasing phase of the second stage bubble, since the bubble radius in the second stage is of same dimensions as in the first stage, therefore further acceleration in the second stage is not possible. In cases (iii) and (iv) the effective acceleration length is short, as well as the laser in the second stage is not strong enough to well guide, therefore electrons are not accelerated in the second stage. Consequently, the two-stage acceleration driven by two-color laser pulses can provide significantly higher energy gain than single color cases.

Figure S1. Time evolution of maximum energy of the accelerating electron bunch in case (i) Comparison among electron energy spectrum obtained in LWFA driven by equal energy (i) TFTP with same parameters same as in Fig. 2, (ii) SFTP with identical laser pulses ($a_{0(\omega_{0})}=4.34$, $W_{0(\omega_{0})}=7c/\omega_{p}$, $\tau_{L(\omega_{0})}=3\omega_{p}^{-1}$ and $\omega_{0}=20\omega_{p}$), (iii) SFTP with leading pulse $a_{0(\omega_{0})}=5.5$, trailing pulse $a_{0(\omega_{0})}=3.835$ and rest of the parameters same as in case (ii), and (iv) SFTP with leading pulse $a_{0(\omega_{0})}=6.0$, trailing pulse $a_{0(\omega_{0})}=1.85$. Time delay between the two pulses in all the cases is $6\omega_{p}^{-1}$.
